# Are SARS-CoV-2 reinfection and Covid-19 recurrence possible? a case report from Brazil

**DOI:** 10.1590/0037-8682-0619-2020

**Published:** 2020-09-18

**Authors:** Lívia Pimenta Bonifácio, Ana Paula Sulino Pereira, Daniel Cardoso de Almeida e Araújo, Viviane da Mata Pasti Balbão, Benedito Antônio Lopes da Fonseca, Afonso Dinis Costa Passos, Fernando Bellissimo-Rodrigues

**Affiliations:** 1 Universidade de São Paulo, Faculdade de Medicina de Ribeirão Preto, Ribeirão Preto, SP, Brasil.; 2 Serviço de Vigilância Epidemiológica Municipal de Ribeirão Preto, Ribeirão Preto, SP, Brasil.; 3 Universidade de São Paulo, Faculdade de Medicina de Ribeirão Preto, Núcleo de Vigilância Epidemiológica Hospitalar do Hospital das Clínicas, Ribeirão Preto, SP, Brasil.

**Keywords:** Covid-19, SARS-CoV-2, Case Report, Reinfection, Recurrence

## Abstract

With the large number of individuals infected and recovered from Covid-19, there is intense discussion about the quality and duration of the immunity elicited by SARS-CoV-2 infection, including the possibility of disease recurrence. Here we report a case with strong clinical, epidemiological and laboratorial evidence of, not only reinfection by SARS-CoV-2, but also clinical recurrence of Covid-19.

## INTRODUCTION

A little less than a year ago, a new coronavirus, called SARS-CoV-2, was identified in a province of China and gave rise to the disease Covid-19, which quickly spread to all continents, generating a pandemic with serious repercussions for global public health^1^ ^-^ ^3^.

One of the many important questions remaining to be answered regarding Covid-19 disease is whether or not SARS-CoV-2 infections induce long-lasting immunity against the virus. Case reports have identified persistent or recurrent elimination of viral RNA in nasopharyngeal samples, raising the possibility of reinfection by SARS-CoV-2^4^ ^-^ ^7^. The present case report describes the investigation of a possible SARS-CoV-2 reinfection, notified to the local Epidemiologic Surveillance Service.

## CASE REPORT

Our patient is a 24-year-old white female who works as a nursing technician. She is overweight and her past medical history included sporadically episodes of headache, but no other chronic diseases. Her vaccines were up-to-date according to the national vaccine recommendations. She denied using continuous medications, smoking, drinking alcohol, or using illicit drugs.

**FIGURE 1:**
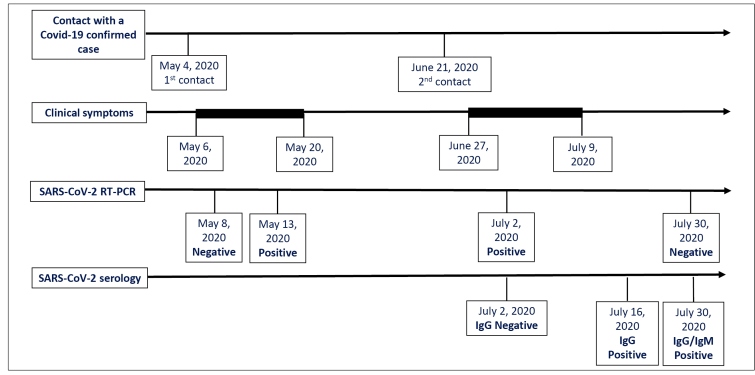
Covid-19 recurrence: timeline of events.

On May 4, 2020, she provided nursing care for a colleague presenting with clinical symptoms of Covid-19. Two days later, she complained of headache, more severe than usual, soon followed by the appearance of malaise, adynamia, feverish sensation, sore throat and nasal congestion. By that time, Covid-19 was confirmed in her colleague, by RT-PCR. On the 2^nd^ day of symptoms, she was submitted to nasopharyngeal and oropharyngeal swab sampling for SARS-CoV-2 RT-PCR which failed to detect the virus RNA. As the symptoms persisted and the contact with a confirmed case was evident, the patient was instructed to repeat the tests (Figure 1).

On the 7^th^ day of symptoms, nasopharyngeal and oropharyngeal samplings were repeated and resulted positive for SARS-CoV-2 RT-PCR (Fragments N1 and N2 of SARS-CoV-2, according to the Centers for Disease Control protocol^8^ ^,^ ^9^). Among her two family members, one developed flu-like symptoms, but he was not tested for diagnostic confirmation. She has been treated as an outpatient and has been prescribed only symptomatic medications, namely naproxen and dipyrone. The patient presented with a mild disease with a complete resolution of symptoms within 10 days. She remained home isolated for 14 days.

Following her recovery, the patient resumed working and spent 38 days completely asymptomatic. On June 21, two family members develop flu-like symptoms and were lately confirmed as Covid-19 cases by RT-PCR. One of these family members was in home isolation, but the other had recently returned to work activities as a taxi driver. On June 27, she woke up with malaise, myalgia, severe headache, fatigue, weakness, feverish sensation, sore throat, anosmia and dysgeusia. The symptoms worsened, with development of diarrhea and coughing in the following days. On the 5^th^ day of symptoms onset, a SARS-CoV-2 RT-PCR was positive on a new nasopharyngeal and oropharyngeal swab sampling. A rapid test for SARS-CoV-2 antibody detection (IgG/IgM, Immunochromatography, Wondfo^TM^) collected on the same day was negative. 

Once more, she did not require hospitalization and has been treated with symptomatic medications, only. The patient experienced spontaneous resolution of most of the acute symptoms in 12 days, but persisted with headache and hyposmia until the date of the present investigation (63 days after the onset of symptoms). She also reported that the doctor who provided medical care for her on the second episode developed flu-like symptoms about a week after the contact, and Covid-19 was lately confirmed on him by means of nasopharyngeal RT-PCR. 

SARS-CoV-2 antibody detection was positive by chemiluminescence (IgG, Chemiluminescence, Abbott^TM^), collected on the 19^th^ and 33^rd^ days after the new onset of symptoms, and by ELISA (IgM, immunoenzymatic assay - ELISA, Vircell^TM^), collected on the 33^rd^ day after symptoms onset. Clinical and laboratory investigation carried out after the two episodes of infection did not identify any evidence of primary or acquired immunodeficiency (Table 1).

**TABLE 1:** Clinical and laboratory findings of the patient with recurrent Covid-19.

	Laboratory parameters on July 30, 2020	
Laboratory tests	Results	Reference values
Blood glucose	70.60 mg/dL	70.0 - 100.0 mg/dL
Anti-HIV ELISA	Not detected	Not detected
IgG serum level	1605 mg/dL	650 - 1600 mg/dL
IgA serum level	527 mg/dL	40.0 - 350.0 mg/dL
IgM serum level	196 mg/dL	50.0 - 300.0 mg/dL
Hemoglobin	12.4 g/dL	12.4 - 16.1 g/dL
Hematocrit	37 %	35.4 - 46.3%
White blood cell	6.7 x 10^3^/µL	4.05 - 11.84 x 10^3^/µL
Neutrophil	3.3 x 10^3^/ µL	1.7 - 7.2 10^3^/µL
Lymphocyte	2.8 x 10^3^/µL	1.17 - 3.45 x 10^3^/µL
Platelets	382 x 10^3^/µL	203 - 445 x 10^3^/µL
C-reactive protein	<0.40 mg/dL	<1.0 mg/dL
AST aspartate aminotransferase	14.0 U/L	< 32.0 U/L
ALT alanine aminotransferase	10.00 U/L	10 - 49 U/L
D-dimer	0.16 µG /mL	≤ 0.5 µG/mL
Urea	25.68 mg/dL	19 - 49 mg/dL
Creatinine	0.69 mg/dL	0.55 - 1.02 mg/dL
	**Clinical parameters on July 30. 2020**	
**Parameter**	**Results**	**Reference values**
Respiratory frequency	12 ipm	16-20 ipm
Oxygen saturation SatO_2_	98%	≥95%
Heart rate	86 rpm	-
Blood pressure	120x60mmHg	-
Pulmonary auscultation	Breath sounds present bilaterally without adventitious sounds	-

## DISCUSSION

Since the beginning of the Covid-19 pandemic, due to several reports of persistent detection of viral RNA by RT-PCR in a nasopharyngeal or oropharyngeal swab, but without recurrence of symptoms, the possibility of SARS-CoV-2 reinfection has been suggested and investigated by different researchers around the world^5^ ^,^ ^6^ ^,^ ^10^ ^,^ ^11^.

A study from the Korean Centers for Disease Control investigating re-positive cases shows that RT-PCRs of cases remained positive for up to 12 weeks. Of those re-positive cases for which symptoms were investigated, 44.7% of patients were symptomatic, but there was no evidence for secondary infections on close contacts of these symptomatic re-positive cases^12^.

There are at least three potential reasons to explain such unusual clinical picture presented by this patient. First of all, there is the possibility of a single persistent infection with recrudescence of symptoms after a while. Although theoretically possible, the fact that she fell ill for the second time about a week after being home exposed to confirmed Covid-19 cases makes this explanation very unlikely.

Another potential explanation is that her second illness could be due to another respiratory virus and the RT-PCR could be only residually positive, or even falsely positive. Again, this is possible but she reported closed contact with a Covid-19 case and not with a flu or common cold case. Moreover, she developed anosmia and dysgeusia which are typical symptoms of Covid-19, and are rarely found in other respiratory tract infections.

 The third explanation is that this a true episode of reinfection by SARS-CoV-2, with clinical recurrence of Covid-19, similarly to a few other cases reported^13^ ^,^ ^14^. Reinfection on this case is supported by epidemiological evidence (contact with confirmed cases in both circumstances, possible transmission to a third person in the second episode), clinical evidence (recurrence of typical symptoms), and laboratorial evidence (positive virological and serological results, chronologically aligned with each overt disease), which fulfill twice the case definition of Covid-19 proposed by the World Health Organization. Since her samples were discarded soon after the tests have been performed, we could not further evaluate the virus genome responsible for each episode of infection. Therefore, we do not know if the reinfection was due to a variant of the original SARS-CoV-2 virus, or she became reinfected by the same virus strain because she did not develop full immunity against it, after the first episode. 

In conclusion, this case report presents strong evidence that SARS-CoV-2 reinfection and Covid-19 recurrence, although rare, are possible. This possibility should be further investigated in patients presenting with recurrence of Covid-19 symptoms.
